# Draft Genome Sequence of *Thermogemmatispora onikobensis* NBRC 111776^T^, an Aerial Mycelium- and Spore-Forming Thermophilic Bacterium Belonging to the Class *Ktedonobacteria*

**DOI:** 10.1128/genomeA.01156-16

**Published:** 2016-10-13

**Authors:** Hisayuki Komaki, Akira Hosoyama, Shuhei Yabe, Akira Yokota, Yoshihito Uchino, Hideaki Takano

**Affiliations:** a Biological Resource Center, National Institute of Technology and Evaluation (NBRC), Chiba, Japan; b NBRC, Tokyo, Japan; c Graduate School of Agricultural Science, Tohoku University, Miyagi, Japan; d Hazaka Plant Research Center, Kennan Eisei Kogyo Co., Ltd., Miyagi, Japan; e College of Bioresource Sciences, Nihon University, Kanagawa, Japan

## Abstract

Here, we report the draft genome sequence of *Thermogemmatispora onikobensis* NBRC 111776^T^, an aerial mycelium- and spore-forming thermophilic bacterium belonging to the class *Ktedonobacteria*. The genome contains five biosynthetic gene clusters coding for secondary metabolites, such as terpene, thiopeptide, lantipeptide, nonribosomal peptide, and lassopeptide, suggesting the potential to produce secondary metabolites.

## GENOME ANNOUNCEMENT

*Thermogemmatispora onikobensis* ONI-1^T^, isolated from fallen leaves on geothermal soils, is a thermophilic, Gram-positive, and sporulating bacterium belonging to the class *Ktedonobacteria* within the phylum *Chloroflexi* (1). The class *Ktedonobacteria* contains six cultured species with validly published names: *Ktedonobacter racemifer* (2), *Thermosporothrix hazakensis* (3), *Thermogemmatispora onikobensis, Thermogemmatispora foliorum* (1), *Thermogemmatispora carboxidivorans* (4), and *Thermosporothrix narukonensis* (5). The complete genome sequence of *Ktedonobacter racemifer* SOSP1-21^T^ has already been reported (6). Since these species form branched vegetative and aerial mycelia, their colony morphologies resemble those of actinomycetes of the phylum *Actinobacteria*. Actinomycetes are widely recognized as rich sources for a variety of bioactive secondary metabolites (7), and secondary metabolism is often associated with morphological developments in microorganisms (8–10). Therefore, members of the class *Ktedonobacteria* are recently expected as new sources, and indeed, new compounds were discovered from *T. hazakensis* SK20-1^T^ (11). The genome project for *T. hazakensis* SK20-1^T^ is also ongoing (12, 13), which suggests that the genome contains many biosynthetic gene clusters coding for secondary metabolites, such as polyketides and nonribosomal peptides (13). In contrast, the diversity of secondary metabolic pathways in the genus *Thermogemmatispora* was unclear, because no genome information of this genus had been available when we began this study. Hence, we conducted genome sequencing of *T. onikobensis* to assess its potential as a secondary metabolite producer.

*T. onikobensis* ONI-1^T^ was deposited into the NBRC culture collection and has been registered as NBRC 111776^T^. The whole genome of *T. onikobensis* NBRC 111776^T^ was sequenced by paired-end sequencing with MiSeq (Illumina; 792-Mb sequences, 142.5-fold coverage). These reads were assembled using Newbler version 3.0 and subsequently finished using GenoFinisher (14), which led to a final assembly of 112 scaffold sequences of >500 bp each. The total size of the assembly was 5,556,501 bp, with a G+C content of 61.1%. Secondary metabolic gene clusters were surveyed using antiSMASH (15). The genome harbors biosynthetic gene clusters for terpene, thiopeptide-lantipeptide, lantipeptide, and lassopeptide, which are encoded in scaffold00005, scaffold00006, scaffold00015, and scaffold00090, respectively. A biosynthetic gene cluster for nonribosomal peptide is also present, but it was not completely sequenced and is divided into scaffold00050, scaffold00083, and scaffold00122.

During this study, the draft genome sequence of *Thermogemmatispora carboxidivorans* PM5^T^ (GenBank accession no. JNIM01000001) was released to the public. The genome of *T. carboxidivorans* PM5^T^ also possesses biosynthetic gene clusters for terpene, thiopeptide-lantipeptide, lantipeptide, and nonribosomal peptide, which are similar to those of *T. onikobensis* ONI-1^T^, suggesting that these four clusters are conserved in the genus *Thermogemmatispora*. In contrast, the lassopeptide biosynthetic gene cluster is specific to *T. onikobensis* ONI-1^T^, since *T. carboxidivorans* PM5^T^ does not harbor any lassopeptide biosynthetic gene clusters.

Here, we publish the draft genome sequence of *T. onikobensis* ONI-1^T^. The genome sequence will provide significant information to elucidate the potential as a secondary metabolite producer.

### Accession number(s).

The draft genome sequence of *Thermogemmatispora onikobensis* NBRC 111776^T^ has been deposited in the DDBJ/ENA/GenBank database under the accession no. BDGT00000000. The version described in this paper is the first version, BDGT01000000.
